# CT-guided placement of microcoil end in the pleural cavity for video-assisted thoracic surgical resection of ground-glass opacity: a retrospective study

**DOI:** 10.1186/s13019-022-02048-6

**Published:** 2022-12-17

**Authors:** Jianli An, Yanchao Dong, Yanguo Li, Xiaoyu Han, Hongtao Niu, Zibo Zou, Jingpeng Wu, Ye Tian, Zhuo Chen

**Affiliations:** 1 Department of Interventional treatment, Qinhuangdao Municipal No. 1 Hospital, No. 258 Wenhua Road, Hebei Province 066000 Qinhuangdao, People’s Republic of China; 2 Department of Riadiology, Qinhuangdao Municipal No. 1 Hospital, No. 258 Wenhua Road, Hebei Province Qinhuangdao, People’s Republic of China; 3 Department of Cardiovascular, Qinhuangdao Municipal No. 1 Hospital, No. 258 Wenhua Road, Hebei Province Qinhuangdao, People’s Republic of China

**Keywords:** Video-assisted thoracic surgery, Ground-glass opacity, CT, Microcoil

## Abstract

**Background:**

The aim of the study was to investigate and summarize the effectiveness and safety of CT-guided microcoil localization before video-assisted thoracic surgery (VATS) for the removal of ground-glass opacity (GGO).

**Methods:**

A total of 147 patients with GGO who were treated at our hospital between January 2019 and February 2021 were retrospectively analyzed. They were divided into two groups according to the final position at the end of the microcoil: intracavity (n = 78) and extracavity (n = 69), which were compared based on puncture complications and influence of the coil end position on VATS.

**Results:**

The proportions of supine and prone positions in the intracavity group were significantly higher than those in the extracavity group (82.1% vs. 66.7%, P < 0.05). The incidence of intrapulmonary hemorrhage, chest pain, and coil displacement in the intracavity group was significantly lower than that in the extracavity group (28.2% vs. 46.4%, 19.2% vs. 39.1%, 1.3% vs. 11.6%, P < 0.05, respectively); however, the incidence of pneumothorax was not significantly different (P > 0.05). The time of VATS and the rate of conversion to thoracotomy in the intracavity group were significantly lower than those in the extracavity group (103.4 ± 21.0 min vs. 112.2 ± 17.3 min, 0% vs. 5.8%, P < 0.05, respectively).

**Conclusion:**

CT-guided placement of the microcoil is a practical, simple, and convenient localization method before VATS, with a high success rate and few complications. Furthermore, it is a better alternative method to place the end of the coil in the pleural cavity because of the lower complication rate, shorter VATS time, and lower rate of thoracotomy conversion.

## Background

Lung cancer is one of the most dangerous malignant tumors with high morbidity and mortality, especially in the elderly [1]. Related studies have shown that the 5-year survival rate of patients with advanced lung cancer is < 2% [2, 3], while the 5-year survival rate of early lung cancer with ground-glass opacity (GGO) was as high as 100% [4, 5]. Therefore, early diagnosis and treatment of lung cancer are particularly important.

With the development of medical imaging technology, high-resolution computed tomography has gradually become popular, and small pulmonary nodules have been increasingly found, thus, attracting attention of researchers [6]. However, in the present case, because the GGO and solid component were relatively small, performing the needle biopsy was challenging. Moreover, there were few tissues available, making it difficult to make an accurate pathological diagnosis, which led to delay in diagnosis. For all small pulmonary nodules, if the possibility of early lung cancer is highly suspected, surgical resection should be the first choice, which aims to achieve prompt diagnosis and treatment [7]. However, traditional thoracotomy is highly traumatic and prone to complications, such as postoperative pain. Video-assisted thoracic surgery (VATS) has gradually replaced traditional thoracotomy because of its advantages of integrated diagnosis and treatment, minimal invasiveness, and less complications [8]. However, previous studies reveal the challenges encountered by the surgeon in performing VATS, including difficulty in locating pulmonary nodules quickly and accurately during surgery, especially for ground-glass nodules with small size, deep location, and few solid components [9]. Studies have shown that it is difficult to locate more than half of the nodules accurately in VATS; thus, thoracotomy is needed [10].

Therefore, the key to completing the operation was to locate the GGO effectively before performing VATS. As early as the 1990s, Asamura et al. first used platinum microcoils applied to vascular embolization to locate GGO, which achieved good results [11]. Subsequently, Powell et al. developed a better positioning method, which placed the end of microcoils in the pleural cavity, so that the position of microcoils could be visually observed during VATS operation, thereby making GGO resection easier to perform [12]. We used this microcoil positioning method from the Powell, wherein the head of coil was placed around the GGO and the end of coil was placed in the pleural cavity, so as to locate the GGO during the VATS for accurate resection. However, the end of the coil cannot be accurately positioned in the pleural cavity, and sometimes, the end may be located in the soft tissue of the chest wall outside the pleura. There has been no previous research on the influence of microcoil tail position on preoperative positioning. Therefore, the purpose of this study was to discuss and review the CT-guided positioning of microcoils for GGO before VATS and the influence of the different positions of the end of microcoils on puncture complications and VATS.

## Methods

### Study subjects

The clinical data of patients with GGOs in our hospital from January 2019 to February 2021 were analyzed retrospectively. The inclusion criteria were as follows: (1) patients diagnosed with solitary GGO on chest CT (lung window setting) and after more than six months of follow-up; (2) the diameter of GGO increased or the solid components increased, identified by more than two radiologists with more than 10 years of experience in detecting suspected malignant nodules; (3) nodule diameter ≤ 15 mm; and (4) according to the shape and position of CT, it was considered that it was unlikely to be touched during the operation. The exclusion criteria were as follows: (1) serious cardiopulmonary dysfunction; (2) abnormal coagulation function; (3) contraindication of VATS; (4) a large vascular structure around the GGO with extremely high risk of puncture; and (5) restlessness or inability to coordinate with the puncture location due to severe cough. This study was approved by the Medical Ethics Committee of Qinhuangdao Municipal No.1 Hospital. Informed consent forms were waived since this was a retrospective study.

**Fig. 1:**
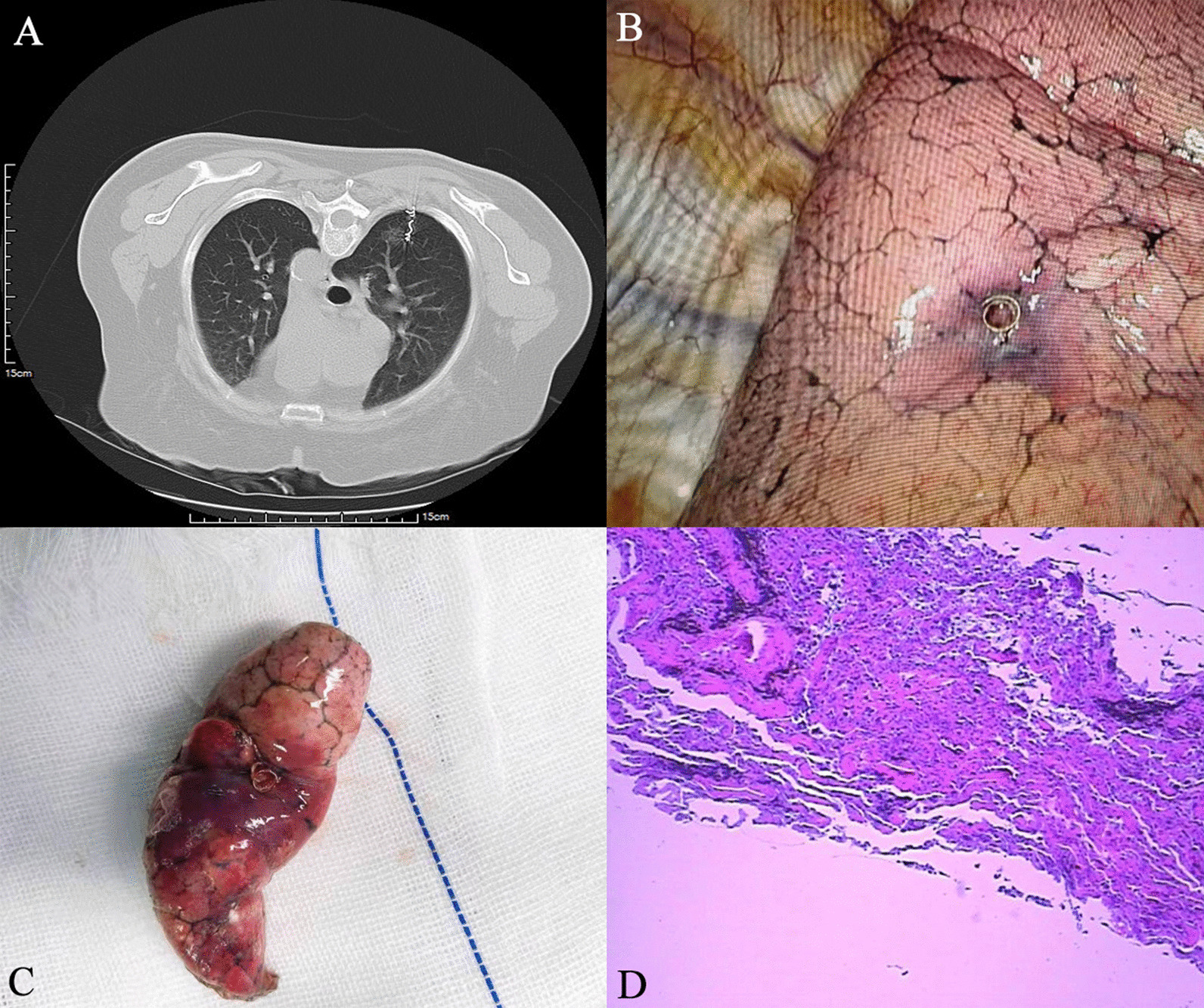
A 61-year-old female patient presented with a 14 mm GGO in the right lower lobe of the lung. The end of the coil was placed in the pleural cavity and a VAST wedge resection was performed, as shown in **A**, **B** showed the end of the coil below the pleural surface, **C** showed the wedge-shaped resection of lung tissue (5 cm*4 cm*3 cm), and **D** showed the patient's pathological findings: preinvasive carcinoma

**Fig. 2:**
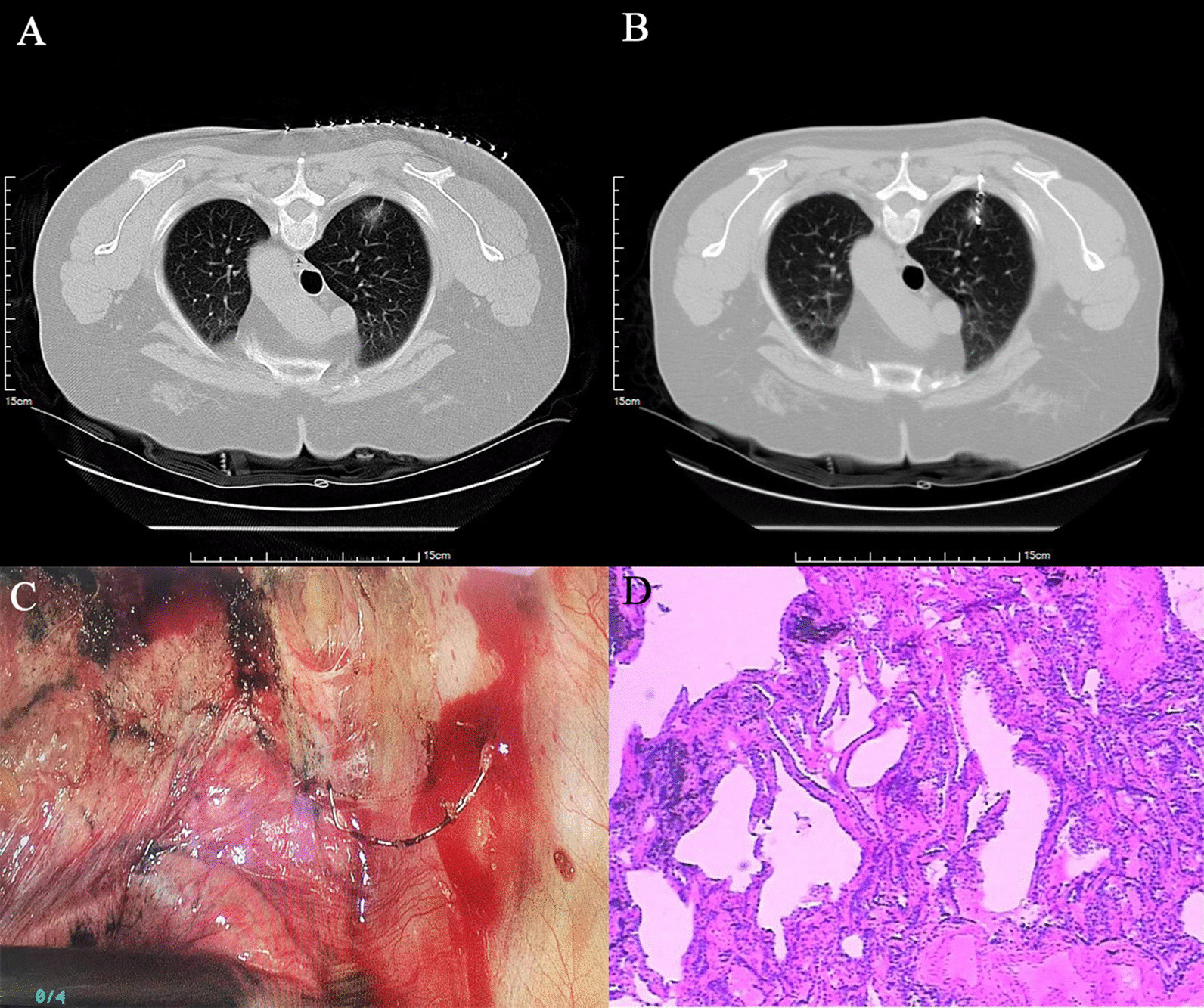
**A** was a 51-year-old woman with a 15-mm mGGO in the upper lobe of the right lung. **B** was the end of the coil placed outside the pleural cavity, in the soft tissue of the chest wall, **C** showed that the end of the coil was still in the chest wall when the lung tissue was removed by VATS. **D** showed the patient’s pathological findings: adenocarcinoma

A total of 147 patients who received CT-guided microcoil positioning GGO before VAST were selected and divided into two groups according to the position of the coil end. Seventy-eight cases were included in the intracavity group (coil end was located in the pleural cavity) (Fig. 1) and 69 patients in the extracavity group (coil end was located outside the pleural cavity and in the soft tissue of the chest wall) (Fig. 2).

### CT-guided microcoil localization

CT-guided microcoils were positioned within 12 h before VATS. The positioning scheme and body position were discussed with thoracic surgeons based on the preoperative CT. CT (interval 1.25 mm) was performed after placement of the locator before puncture, and the position of the puncture point, depth, and angle of needle insertion were determined to avoid intercostal nerves, blood vessels, large blood vessels, and the bronchi. The step-by-step preparation for the procedure were as follows: Routine disinfection and towel laying were done, local infiltration anesthesia with 2% lidocaine was administered, the patient was instructed to hold breath and a 21 G Chiba needle was inserted into the lung pleura, CT was quickly re-examined, the patient was again instructed to hold breath then the needle tip position was adjusted, the needle was inserted into the normal lung tissue around the lesion (< 5 mm distance from the GGO), CT was re-examined to determine the position, and then, the needle core was pulled out and the microcoil (COOK) was connected. We selected a microcoil with the appropriate length according to the pleural distance of the nodules. Generally, it should be at least > 2 cm than the distance from the nodule to the pleura. The coil was pushed around the nodule for anchorage, then the plain CT scan was repeated. The needle was gradually withdrawn according to the measured distance between the needle tip and the lung pleura, then, the end of the microcoil was released to form a ring outside the visceral pleura. After observing for approximately 10 min, the CT was re-checked to determine the position of the coil and to monitor for intrapulmonary hemorrhage and pneumothorax. The patient was transferred back to the ward without signs of chest tightness, shortness of breath, or bleeding at the puncture point. The costs of coil implantation was less than $500.

### VATS resection

In all cases, thoracoscopic resection was performed within 12 h after microcoil placement. All patients were transported by bed to a pre-operative holding area before being transferred to the operating room. Patients were instructed to avoid strenuous activities, panting, and coughing, as much as possible.

Intravenous general anesthesia was administered via double-lumen endotracheal intubation, with the healthy side placed in the 90∘lateral position, and the towel used was routinely disinfected. The 7th/8th intercostal space of the affected side was selected as the observation hole, and the 3rd/4th intercostal space was selected as the operation hole. After lung collapse, a coil was clearly observed on the surface of the lung. After touching the coil located next to the lesion, a wedge-shaped lobectomy was performed using an Echelon endoscope linear-cutting stapler (Johnson & Johnson, 60 mm). The lung tissue was resected and lesions were found along the microcoils, which were sent for pathological examinations using rapid frozen sections. The decision to undergo lobectomy plus lymph node dissection was based on the results of the pathological examination.

### Data collection

**Fig. 3:**
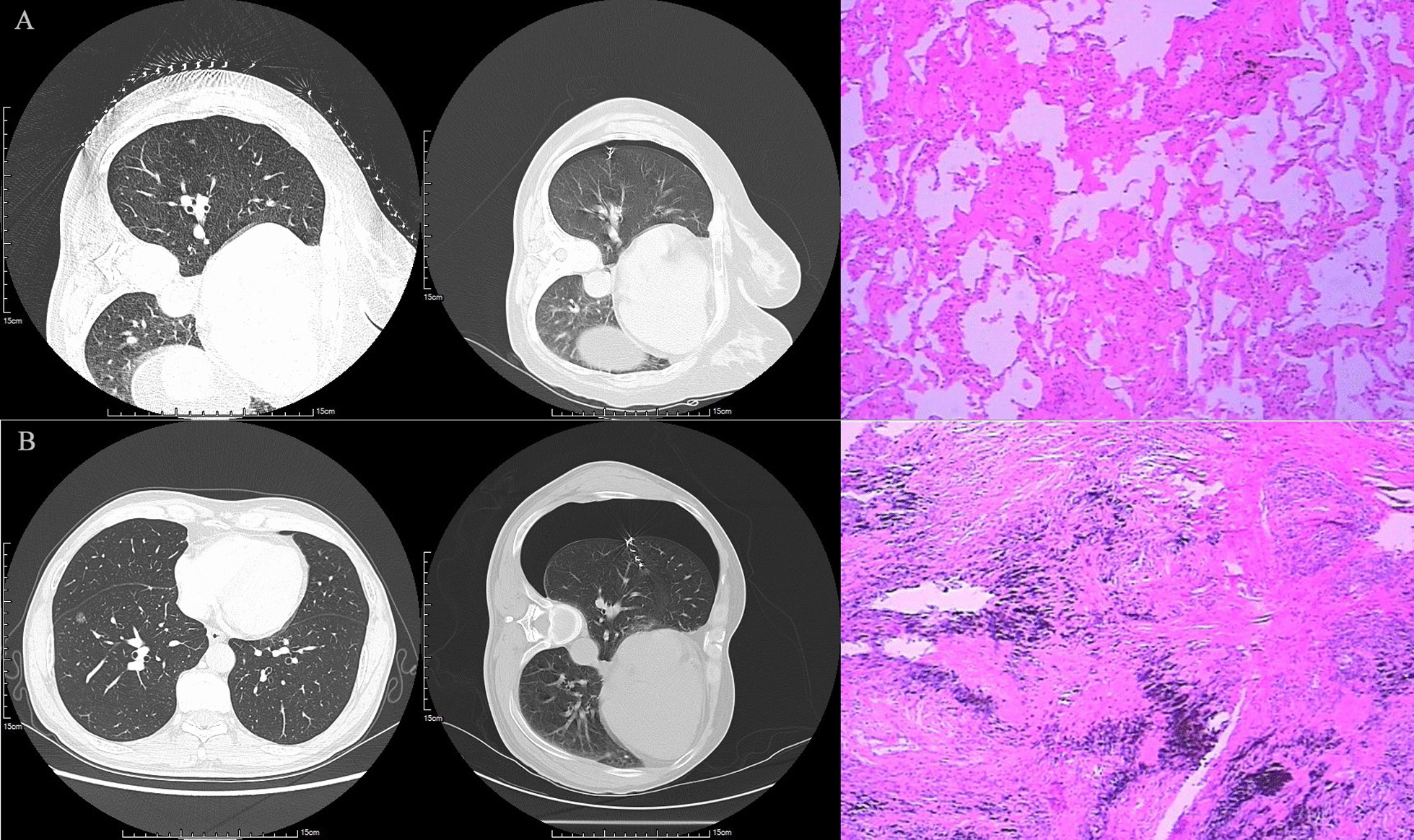
**A** showed a 58-year-old woman with a 5 mm pGGO in the lower lobe of her right lung. Mild pneumothorax appeared after puncture. The distance between visceral pleura and the chest wall was no more than 2 cm. The final pathological result was adenocarcinoma; **B** showed a 51-year-old male with a 9 mm pGGO in the right lower lobe. Severe pneumothorax occurred after the puncture. The visceral pleura was more than 2cm away from the chest wall. The final result showed microinvasive adenocarcinoma

**Fig. 4:**
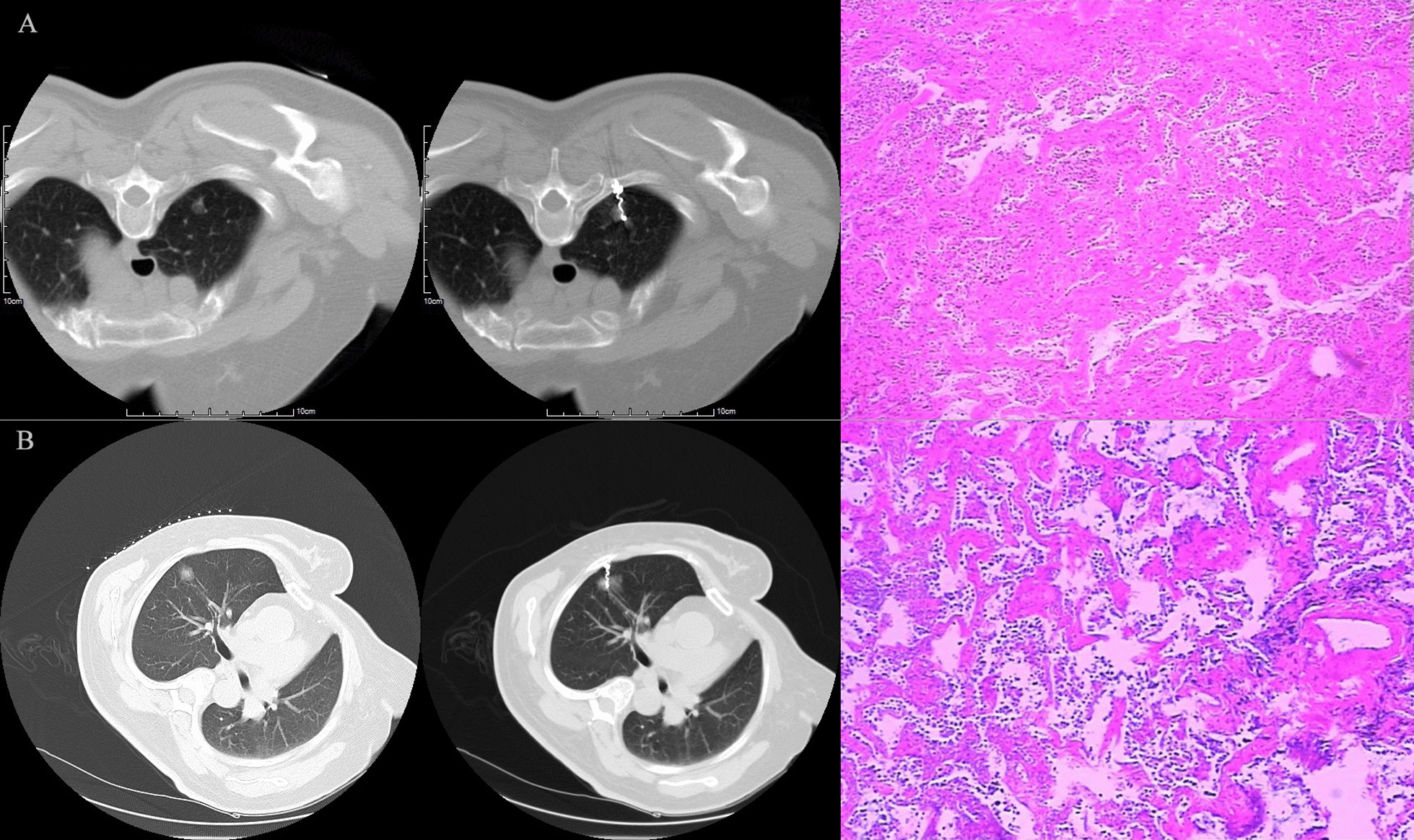
**A** showed a 58-year-old female with an 8 mm pGGO in the right upper lung. There was a small amount of bleeding after puncture. A new ground-glass lesion was seen around the top of the coil in the lung. The original GGO could still be seen, the pathological findings were: invasive adenocarcinoma; **B** showed a 65-year-old woman with a 1 cm-diameter pGGO in the right upper lung, the pathological finding was invasive adenocarcinoma

**Fig. 5:**
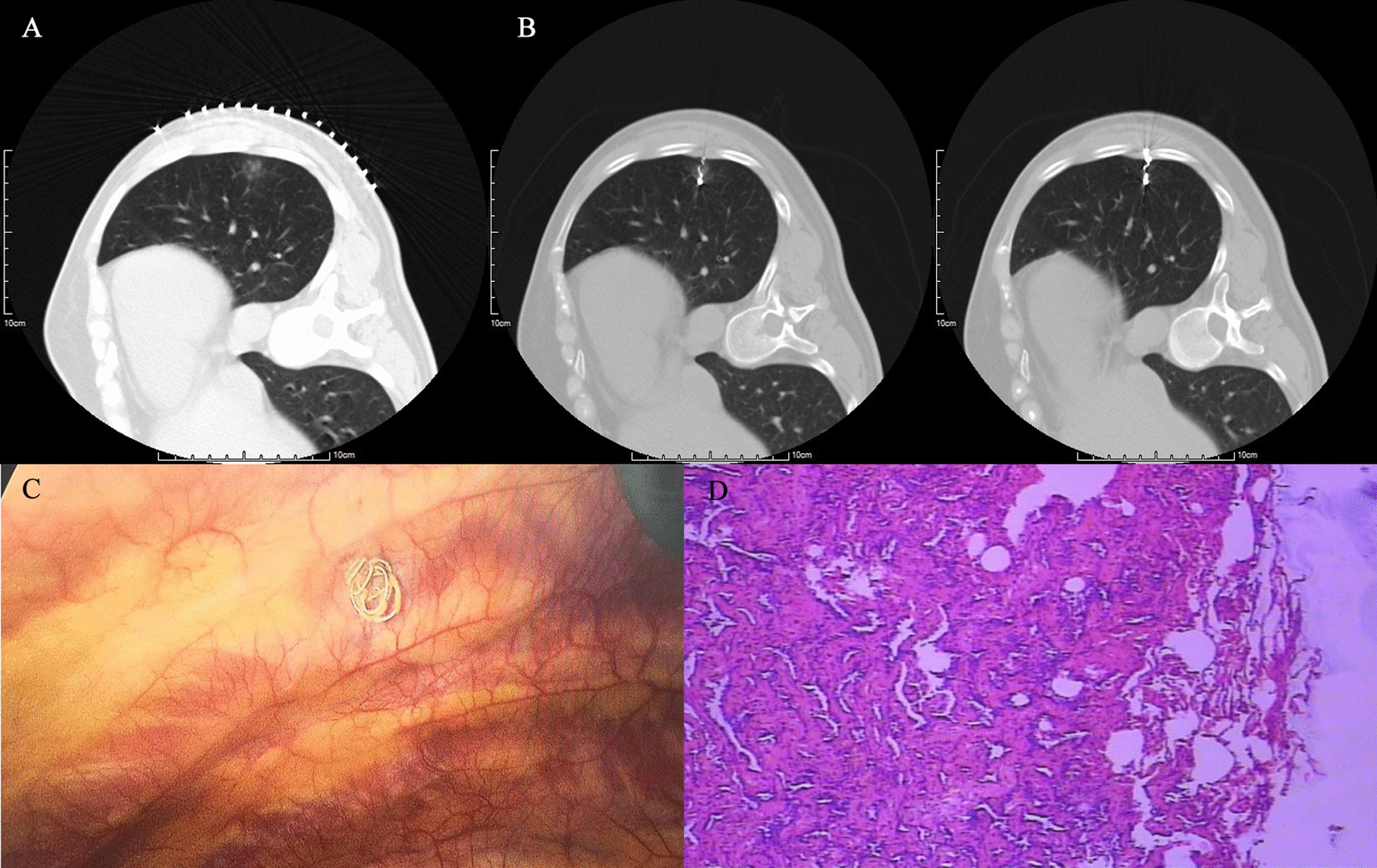
**A** shows a 56-year-old female with an 8 mm pGGO in the left lower lung, **B** showed the end of the coil lodged in the soft tissue of the chest wall, and **C** showed VATS was performed to remove the lung tissue. The coil was released from the lung tissue and lodged in the chest wall. The patient was eventually converted to thoracotomy, the pathological finding was adenocarcinoma

The CT-guided puncture position-related indicators were as follows: patient puncture position, puncture times, positioning time, distance between the head of the coil and the nodule, and positioning success rate. The incidence of puncture complications were described. The type of pneumothorax was categorized as follows: (1) mild pneumothorax: < 2 cm distance between the widest part of the gas density shadow between the affected chest wall and the lung edge; (2) severe pneumothorax: > 2 cm distance between the widest part of the gas density shadow between the affected chest wall and the lung edge. Intrapulmonary hemorrhage was identified as either mild (new patchy ground glass density shadow in the lung; Fig. 3) or severe (new patchy ground-glass density shadow in the lung with inability to show the original pulmonary nodules; Fig. 4). Pain was assessed using the visual analog scale (VAS) (painless = 0, mild pain = 1–3, moderate pain = 4–6, severe pain = 7–10). Furthermore, it was observed whether the coil was displaced during VATS (no displacement: coil shedding; Fig. 5).

The VATS operation index included the following: operation time, conversion to thoracotomy rate, one-time nodule resection rate (one wedge resection defined as complete nodule resection with a sufficient margin range), wedge resection lung tissue volume (length × width × height of lung tissue).

Pathological examination results described the detection rate of lung cancer, atypical tumor-like hyperplasia, hamartoma, inflammatory lesions, and other diseases.

### Statistical analysis

SPSS software (version 18.0) was used for the statistical analysis. The measurement data of normal distribution were expressed as means ± standard deviation (SD), and comparisons between groups were made using the t-test. Frequency and percentage were used for statistical description of the counting data, and the χ^2^ test was used for comparison between groups. Statistical significance was set at P < 0.05.

## Results

### Comparative analysis of basic information

A total of 147 patients with GGO were enrolled, including 78 patients in the intracavity group, with 44 females and 34 males, with an average age of 58.7 ± 11.8 years, ranging from 32 to 79 years old, and an average nodule diameter of 5.02 ± 1.15 mm. There were 33 patients with pure GGO (pGGO) and 45 patients with partial solid GGO (mixed GGO, mGGO). The average distance between the nodules and pleura was 3.03 ± 1.75 cm. In the extracavity group, there were 69 cases, including 40 females and 29 males, with an average age of 56.3 ± 10.0 years, ranging from 34 to 81 years, and an average nodule diameter of 5.07 ± 1.17 mm. There were 31 and 38 patients with pGGOs and mGGO, respectively. The average distance between the nodules and pleura was 2.94 ± 1.46 cm. There was no statistical difference in the basic information between the two groups. Table 1 presents the complete demographic profile of the participants.

**Table 1:** Comparative analysis of basic information of two groups of patients

		Intracavity group (n = 78)	Extracavity group (n = 69)	Statistic		p value
Age		58.7 ± 11.8	56.3 ± 10.0	t = 1.335	0.184	
F/M		44/34	40/29	χ^2^ = 0.036	0.849	
Nodule diameter (mm)		5.02 ± 1.15	5.07 ± 1.17	t = 0.243	0.808	
Nodule nature	pGGO	33	31	χ^2^ = 0.102	0.749	
	mGGO	45	38			
Nodule position	Upper lobe of left lung	19	17	χ^2^ = 0.166	0.997	
	Lower lobe of left lung	17	16			
	Upper lobe of right lung	26	23			
	Middle lobe of right lung	3	3			
	Lower lobe of right lung	13	10			
Distance from nodule to pleura (cm)		3.03 ± 1.75	2.94 ± 1.46	t = 0.343	0.732	

Values were shown as counts or means ± standard deviations

### Comparative analysis of related indexes of the CT-guided puncture

The proportion of patients in supine and prone position in the intracavity group was significantly higher than that in the extracavity group (82.1% vs. 60.9%), while the proportion of patients in lateral position in the intracavity group was significantly lower than that in the extracavity group (17.9% vs. 39.1%). However, there was no significant difference between the two groups in puncture time, puncture positioning time, distance between the coil head and nodule, and positioning success rate (Table 2).

**Table 2:** Comparative analysis of related indexes of CT-guided puncture positioning between two groups of patients

		Intracavity group(n = 78)	Extracavity group(n = 69)	Statistic	p value
Puncture position	Supine position	40	24	χ^2^ = 8.481	0.037^*^
	Prone position	24	18		
	Left lateral position	9	18		
	Right lateral position	5	9		
Puncture times	1	55	50	χ^2^ = 0.132	0.936
	2	20	16		
	3	3	3		
Positioning time (min)		19.56 ± 3.94	18.75 ± 3.35	t = 1.335	0.184
Distance between head of coil and nodule (mm)		1.72 ± 0.45	1.76 ± 0.44	t = 0.566	0.572
Position success rate (%)		100	100	–	–

^*^P value is less than 0.05, showing statistical difference.

Comparing and analyzing the complications after puncture positioning between the two groups, the results showed that the incidence of intrapulmonary hemorrhage, chest pain and coil displacement in the intracavity group was significantly lower than that in the extracavity group (intrapulmonary hemorrhage: 28.2% vs. 46.4%; chest pain:19.2% vs. 39.1%; microcoil displacement: 1.3% vs. 11.6%); However, there was no significant difference in the incidence of pneumothorax between the two groups (26.9% vs. 29.0%) (Table 3).

**Table 3:** Comparison of CT puncture complications between two groups

		Intracavity group(n = 78)	Extracavity group(n = 69)	Statistic	p value
Pneumothorax	Mild	18	18	χ^2^ = 0.077	0.781
	Severe	3	2		
	No	57	49		
Intrapulmonary hemorrhage	Mild	17	29	χ^2^ = 5.202	0.023^*^
	Severe	5	3		
	No	56	37		
Chest pain	Mild	12	19	χ^2^ = 7.104	0.008^*^
	Moderate	3	7		
	Severe	0	1		
	No	63	42		
Microcoil displacement	Drop	1	8	χ^2^ = 6.774	0.013^**^
	No	77	61		

^**^Fisher’s exact probability method was adopted, P value is less than 0.05, showing statistical difference; ^*^P value is less than 0.05, showing statistical difference

### Comparison of VATS operation indexes

Comparative analysis of VATS operation indices between the two groups showed that the VATS time and conversion rate of thoracotomy in the intracavity group were significantly lower than those in the extracavity group. However, there was no significant difference between the one-time resection rate of the nodules and the volume of lung tissue after wedge resection (Table 4).

**Table 4:** Comparative analysis of VATS operation indexes

	Intracavity group(n = 78)	Extracavity group(n = 69)	Statistic	p value	
Time of VATS	103.4 ± 21.0	112.2 ± 17.3	t = 2.761	0.007^*^	
The rate of conversion to thoracotomy	0(0%)	4(5.8%)	χ^2^ = 4.648	0.046^**^	
One-time resection rate of nodules	100%	100%	–	–	
Excised lung tissue volume (cm ^3^)	127.05 ± 58.24	141.26 ± 53.11	t = 1.538	0.126	

^**^Fisher’s exact probability method was adopted, P value is less than 0.05, showing statistical difference; ^*^P value is less than 0.05, showing statistical difference

### Comparison of pathological examination results

Comparison of the pathological examination results between the two groups showed that there was no significant difference in the proportion of lung cancer, atypical adenomatous hyperplasia (AAH), hamartoma, and inflammatory lesions, as shown in Table 5.

**Table 5:** Pathological report

	Intracavity group (n = 78)	Extracavity group (n = 69)	Statistic	p value
Cancer	49	43	χ^2^ = 3.649	0.302
AAH	21	22		
Hamartoma	2	3		
Inflammatory lesions	6	1		

Fisher’s exact probability method was adopted

## Discussion

### Current research situation of puncture location

With the widespread application of low-dose CT, the detection rate of GGO has increased significantly. Clinically, it is necessary to determine the benign or malignant nature of the tumor before further treatment. However, owing to the small GGO, clinical needle biopsy and imaging cannot clearly and accurately study nodules qualitatively. The Fleischner Society GGO treatment guidelines suggest that isolated pGGO and mGGO with a diameter greater than 6 mm should be reexamined within two periods, after 3–6 months and at 6–12 months. If the lesion is enlarged or the lesion density is increased, surgical treatment should be performed, and uniportal VATS wedge resection of lung tissue and segmental and subsegmental pneumonectomy are recommended [13]. Studies have shown that approximately 50% of solitary pulmonary nodules are malignant and should be treated early to prevent further deterioration [14]. Studies have shown that the GGO of stage IA lung adenocarcinoma is more than 50% on CT images, and the prognosis of GGO is better than that of solid nodules after resection, with a 5-year overall survival rate of up to 97% [15]. A recent multicenter study from Japan showed that the 5-year relapse-free survival rate of GGO patients after resection was 99.7% [16]. Therefore, VATS provides a new choice for the early treatment of pulmonary nodules, and the accurate positioning of pulmonary nodules is an urgent problem to be solved [17]. At present, localization methods for pulmonary nodules have diversified, including the imaging localization method, injecting liquid material-mediated localization method [18], and percutaneous placement of solid materials [19]. Each localization method has its advantages and disadvantages. Imaging positioning methods include intraoperative ultrasonic positioning [20], near-infrared fluorescence imaging positioning [21], and surgical navigation puncture robot systems [22]; however, the requirements for equipment and the technical level of operators are high, making it difficult to be widely popularized. However, injection of liquid material-mediated localization methods, including injection of lipiodol [23], methylene blue [24], medical glue [25], indocyanine green fluorescent agent [26], and other liquids, has some shortcomings, such as short retention time, easy diffusion, and uncontrollable injection dose, thus, it is not widely use by thoracic surgeons.

However, percutaneous placement of solid materials was similar to injection of liquid material-mediated localization, which needed to puncture the vicinity of nodules under the guidance of CT and other auxiliary equipment, and place or inject corresponding markers in order to find and accurately locate nodules during operation. Solid materials include hook wires [27], microcoils [28], and other materials. Hook-wire was special for breast nodule positioning [29] because there was no special instrument for lung nodule positioning before in the world, hook-wire was used for lung nodule positioning. Moreover, the hook-wire was quite hard, which increased the risk of pneumothorax, intrapulmonary hemorrhage, and chest pain. However, the microcoil had good flexibility and elasticity, and the spiral structure did not affect the expansion of the surrounding lung tissue to compress the puncture point, thus, reducing the occurrence of the above complications [30]. In addition, the artificial fiber hairs on the surface of the microcoils can increase the friction force so that the microcoils are firmly fixed in the lung tissue, and the displacement and shedding are reduced [17]. Asamura et al. first reported that microcoils were used to locate pulmonary nodules before VATS [11]. In recent years, several studies have confirmed the effectiveness of this method [31, 32].

In the past, most microcoils were placed around the nodules, and pleural displays still cannot directly show the location of the nodules. Presently, more researchers prefer to locate nodules by placing the end of the microcoil in the pleural cavity, which can clearly show the location of coils in VATS and has obvious advantages over previous methods. In this study, we retrospectively analyzed the application of this method for localization. The results showed that 147 patients with GGO were located preoperatively in the two groups, and the total success rate of nodule localization was 100%, with a low incidence of total complications (pneumothorax: 27.9%, intrapulmonary hemorrhage: 36.7%, chest pain: 28.6%, and coil displacement: 6.1%).

### Baseline characteristics

The results showed that there was no statistical difference between the two groups in age, sex composition, nodule diameter, nodule nature, nodule location, and the distance between the nodule and pleura; hence, the two groups were comparable. The results showed that the average age of patients in both groups was over 55 years, and the sex composition showed that female patients were older than male patients (57.1% vs. 42.9%). There were multiple reasons for patient selection. First, elderly women over 55 years old in China had a higher proportion of lung cancer screening, and high-resolution CT found that pGGO and mGGO were higher in female patients [33]. Second, studies by foreign scholars showed that women over 50 years old were risk factors for judging the malignancy of pulmonary nodules [34, 35]. Therefore, women aged > 55 years were selected for this study.

However, the average diameter of the nodules in both groups was > 5 mm, and the proportion of mGGO was slightly higher than that of pGGO (56.5% vs. 43.5%). These patients were selected because pGGO and mGGO with diameters > 5 mm were first reviewed after three months. If the lesion was enlarged or the lesion density was increased, the proportion of malignant lesions that required surgical treatment was higher; therefore, the proportion of patients with mGGO was higher. However, data such as nodule location and distance from the nodule to the pleura were not mentioned in many prediction models, which may have affected the judgment of benign and malignant diseases.

### Puncture position on the position of the end of the coil

A comparison between the two groups showed that the ratio of supine and prone positions in the intracavity group was 82.1%, which was significantly higher than that in the extracavity group (66.7%). There were no significant differences in other puncture times, positioning time, distance between the head of the coil and the nodule, and puncture success rate. Therefore, we considered that the end of the coil was finally placed inside or outside the pleural cavity to a certain extent, which may be related to the patient’s puncture position. We suspected that when the patient was in the supine and prone positions, the body was relatively fixed, so it was difficult to move the position. Therefore, in the process of gradually withdrawing the needle according to the measured distance between the needle tip and pleura, the needle withdrawal distance was accurate, and it was difficult to place the tail end outside the pleural cavity. On the contrary, when the patient adopts the lateral position, it is easy to move slightly, which leads to an inaccurate needle withdrawal distance, and the tail end of the spring coil may be located outside the pleural cavity.

In addition, we found that the positioning time of the intracavity group was slightly shorter than that of the extracavity group, and the distance between the head of the coil and nodule in the intracavity group was slightly lower than that in the extracavity group. Although there was no statistical difference, we speculated that the proportion of patients who might take the lateral position in the extracavity group was higher than that in the intracavity group, which led to the patient’s intolerance of puncture, causing a slight shortening of the positioning time and a minimal increase in the distance between the coil and the nodule. Therefore, a more stable body position for puncture positioning may be a better choice.

### Proportion of pulmonary hemorrhage, chest pain and coil displacement

The total incidence of complications after CT-guided puncture, including pneumothorax (27.9%), pulmonary hemorrhage (36.7%), chest pain (7.5%), and coil displacement (6.1%), was lower in both groups. Compared with previous studies, the incidences of pneumothorax, bleeding, and moderate and severe pain in the Hookwire positioning method were 48.5%, 24.2%, and 24.2%, respectively. Our research results showed that the incidence of bleeding was slightly higher than that in the hookwire positioning method, while the incidence of other complications was significantly lower than that in the hookwire positioning method. The incidence of complications was significantly lower with the microcoil positioning method than with the hookwire positioning method. Compared with other centers using microcoils, the incidences of pneumothorax, bleeding, and moderate and severe pain was 15.2%, 7.6%, and 6.3%, respectively, which were also significantly lower than our experimental results. The possible reason was that the nodules selected by other centers were superficial according to the pleural position, which was less than 1 cm, while our nodules were approximately 3 cm away from the pleura, which was significantly higher than previous research results. Therefore, it may lead to more puncture paths passing through the pulmonary vessels, and the incidence of bleeding was slightly higher than that in the previous experimental results. In addition, the average age of our patients was higher than that of the patients in the above study, which may have led to a slightly higher incidence of complications than the previous experimental results. Another study selected patients with an average age of > 60 years, and the incidence of pneumothorax was as high as 60% [36], which was significantly higher than our experimental results, which may support our conjecture.

However, the comparison of the incidence of complications after puncture between the two groups showed no statistical difference in the incidence of pneumothorax. We considered that the puncture needle was thin, which caused less damage to the pleura, and whether the end of the microcoil was placed inside or outside the pleural cavity, it could block the puncture point to a certain extent, which would not lead to pneumothorax, so there was no significant statistical difference in the incidence of pneumothorax between the two groups. However, the proportion of bleeding, pain, and coil displacement after puncture in the extracavity group was significantly higher than that in the intracavity group. First, we considered that the proportion of patients in the extracavity group who used the lateral position for puncture was higher than that in the intracavity group. The patients in the extracavity group may have had slight displacement, that could have led to needle displacement, which resulted in vascular damage around the nodules and a small amount of bleeding. Second, the end of the coil was placed in the soft tissue of the chest wall, so the coil had certain elasticity and may rebound. During the rebound process, the coil may damage small blood vessels in the puncture path and cause slight bleeding. Because of the high incidence of pain, we considered that the end of the coil was placed in the soft tissue of the chest wall and the head was placed in the lung tissue. When there is relative movement between the two parts, the coil rubs against the pleura, which may stimulate the pleura and cause a high incidence of pain, which can also explain the easy displacement of the coil. Although these complications occurred to some extent, according to the American interventional radiology complication management standard, no further treatment was needed [36].

### VATS time and the rate of conversion to thoracotomy

The VATS time and rate of conversion to thoracotomy were significantly lower in the intracavity group than in the extracavity group. In the extracavity group, they needed to remove the coil on the chest wall to the pleura and then performed wedge resection similar to that in the intracavity group, which may have increased the VATS operation time. Another reason may be related to easy displacement of the coil head, so that when the coil shifts or even falls off, it is difficult to accurately locate the nodule through the coil during the operation. In addition, if the nodules with fallen coils and a deep distance from the pleura cannot be accurately located, they may be converted to an open chest for surgery. Therefore, the rate of conversion to thoracotomy in the extracavity group was significantly higher than that in the intracavity group. However, the total conversion rate of thoracotomy was 2.7%, which was within the acceptable range [37, 38].

The results showed that the volume of lung tissue after wedge resection in the extracavity group was slightly higher than that in the intracavity group, which may also be related to the displacement of coils or transfer to thoracotomy; however, there was no statistical difference, which requires further studies. Nodules in both groups were resected at one time, which avoided the risk of reoperation and reduced the physiological and economic burden on patients. Therefore, CT-guided positioning of the microcoils is of great significance for VATS surgery.

### Pathological results

Our results showed that the proportion of patients with malignant lung tumors diagnosed by pathology was 62.6%, including carcinoma in situ, minimally invasive adenocarcinoma, adenocarcinoma, and squamous cell carcinoma. This was similar to the results of previous studies that showed the malignancy rate of GGO. GGO with highly suspected malignant lesions should be resected by VATS as soon as possible.

## Conclusion

CT-guided positioning of microcoil pulmonary nodules is a practical, simple, and convenient positioning method with a high success rate and few complications. The best position was the supine or prone position, and placing the end of the coil in the pleural cavity had a greater auxiliary effect on VATS operation, with a lower incidence of complications, shorter operation time, and lower rate of conversion to thoracotomy. Therefore, placing the end of the coil in the thoracic cavity before VATS is an ideal positioning method. However, this experiment also has limitations. First, this study adopts the method of retrospective study, thus, it is recommended to conduct prospective researches in the future and reduce recall bias. Second, the selected cases were patients who underwent surgery, and the decision to undergo surgery depended on doctors’ experience and patients’ wishes, which could have contributed to bias. Finally, this study utilized a single-center, with a small sample size. It is imperative to increase the sample size to accommodate the increase in research factors, which includes expansion of the time range of research objects.

## Data Availability

The datasets generated during and/or analysed during the current study are available from the corresponding author on reasonable request.
